# Supplementary figures and images for: Shc1 cooperates with Frs2 and Shp2 to recruit Grb2 in FGF-induced lens development
Source: eLife. 2025 May 6;13:RP103615. doi: 10.7554/eLife.103615 (PMC12055001; doi:10.7554/eLife.103615)

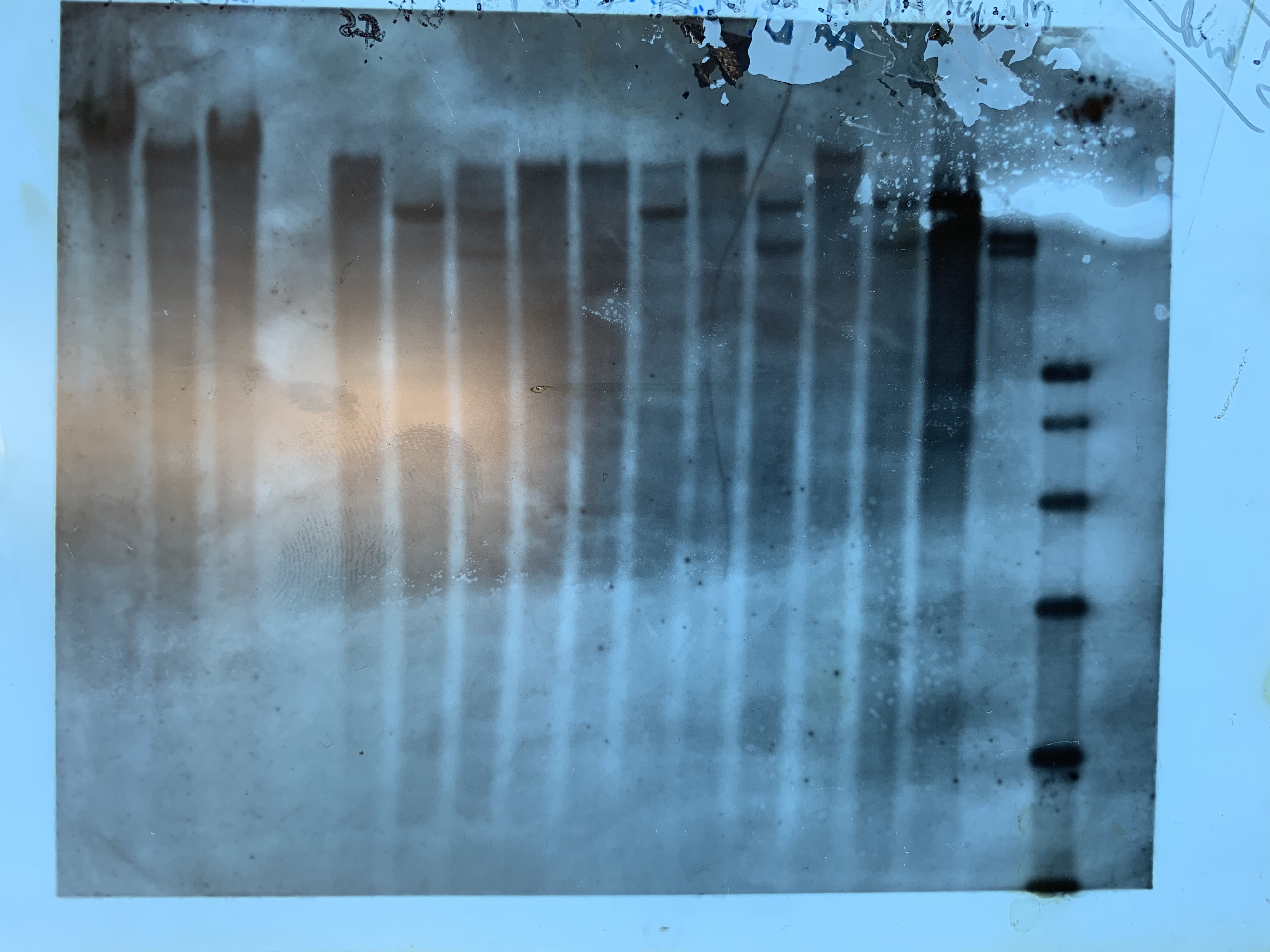

Supplement: Figure 5—source data 1. [file elife-103615-fig5-data1.zip › Shp2YF-5'.JPG]

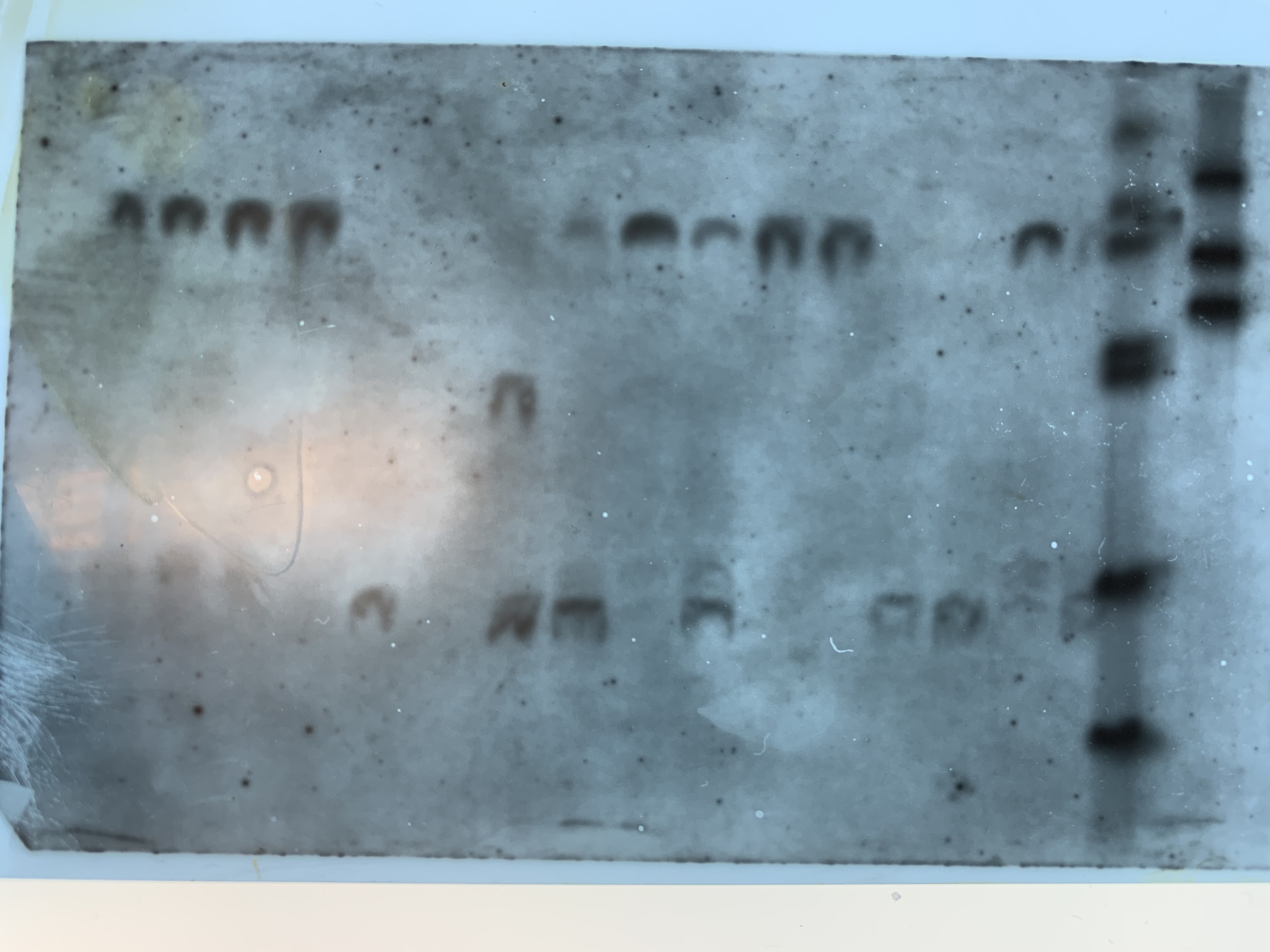

Supplement: Figure 5—source data 1. [file elife-103615-fig5-data1.zip › Shp2YF-3'EcoRV.JPG]

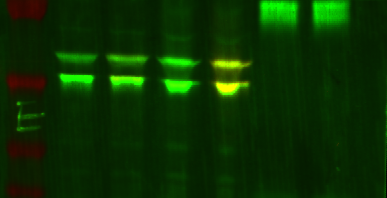

Supplement: Figure 5—source data 3. [file elife-103615-fig5-data3.zip › PDGF pERK.tif]

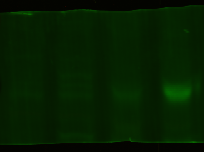

Supplement: Figure 5—source data 3. [file elife-103615-fig5-data3.zip › FGF pShp2.tif]

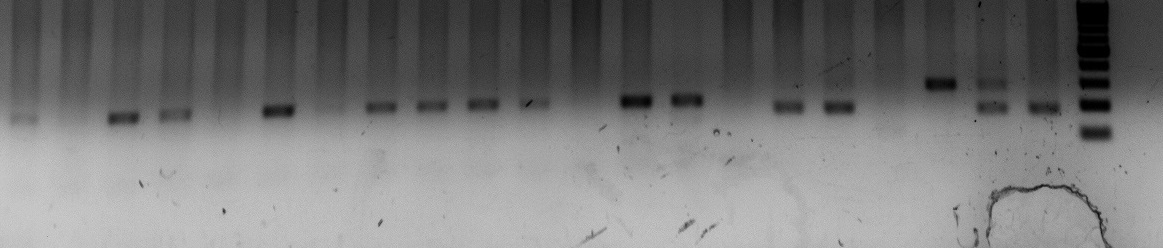

Supplement: Figure 6—source data 1. [file elife-103615-fig6-data1.zip › GY.JPG]

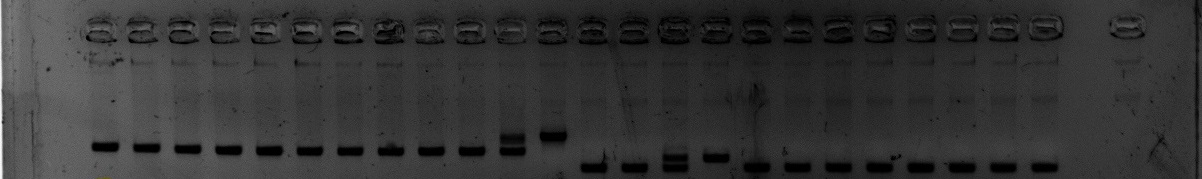

Supplement: Figure 6—source data 3. [file elife-103615-fig6-data3.zip › Sc.JPG]

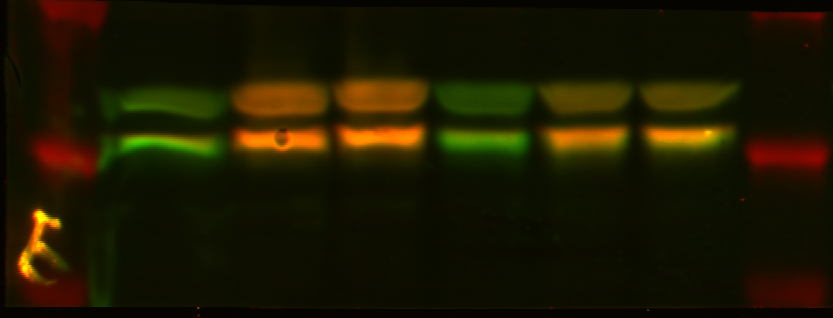

Supplement: Figure 6—source data 5. [file elife-103615-fig6-data5.zip › Shp2CS pERK.tif]

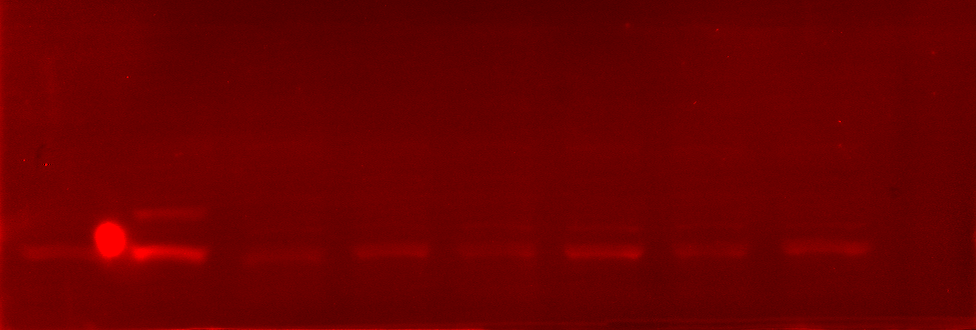

Supplement: Figure 7—source data 1. [file elife-103615-fig7-data1.zip › pShp2.tif]

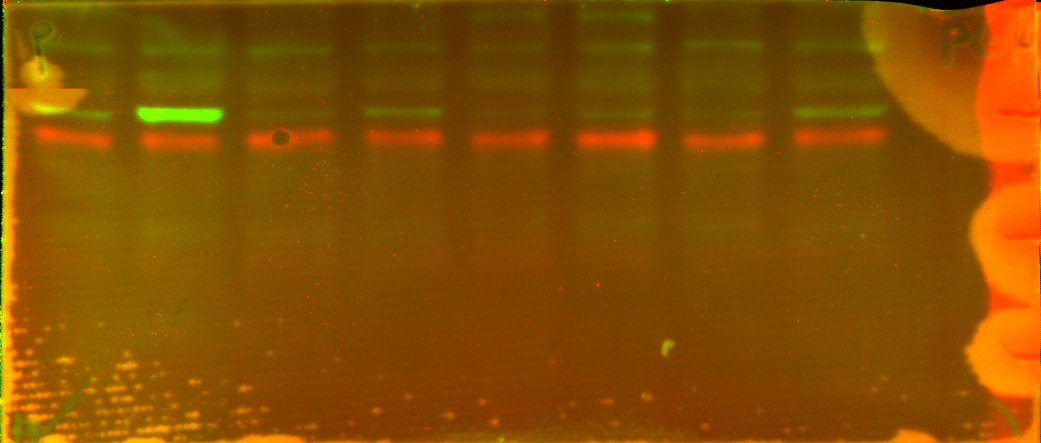

Supplement: Figure 7—source data 1. [file elife-103615-fig7-data1.zip › pCrk.tif]

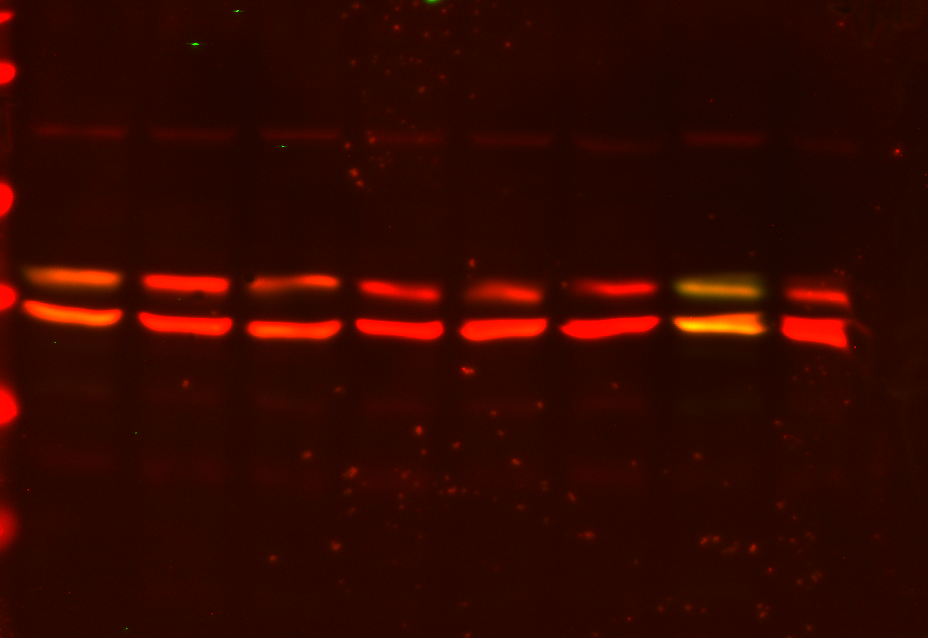

Supplement: Figure 7—source data 1. [file elife-103615-fig7-data1.zip › pERK-ERK.TIF]

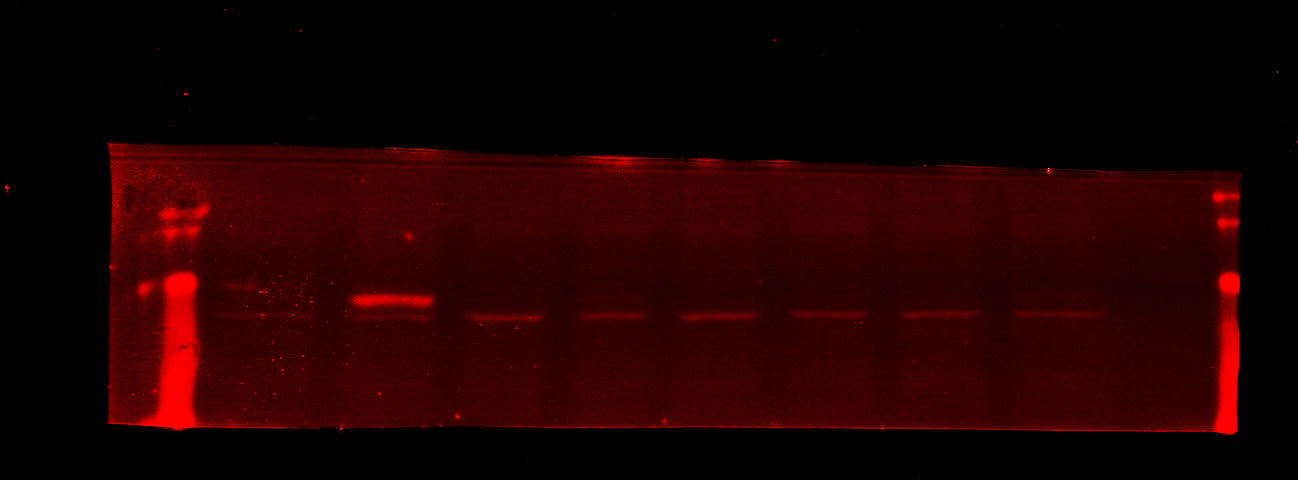

Supplement: Figure 7—source data 1. [file elife-103615-fig7-data1.zip › pFrs2.tif]

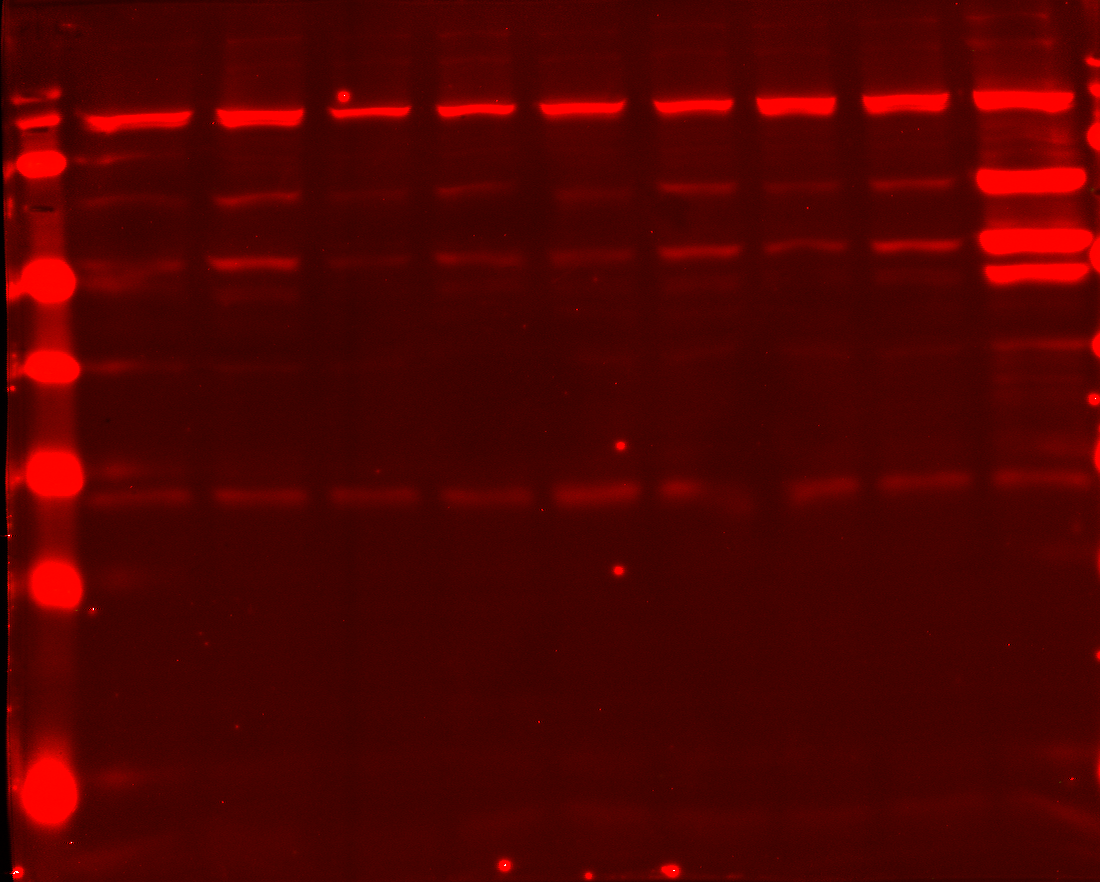

Supplement: Figure 7—source data 1. [file elife-103615-fig7-data1.zip › pShc.tif]
