# Supplementary material for: Shc1 cooperates with Frs2 and Shp2 to recruit Grb2 in FGF-induced lens development
Source: eLife. 2025 May 6;13:RP103615. doi: 10.7554/eLife.103615 (PMC12055001; doi:10.7554/eLife.103615)
Supplement: Figure 5—source data 2. [file elife-103615-fig5-data2.zip › Figure 5C Source data 2.pdf]

5’ Probe

+/+ YF/+

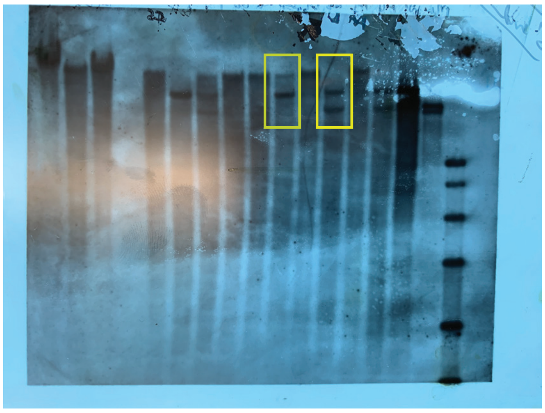

3’ Probe

+/+ YF/+

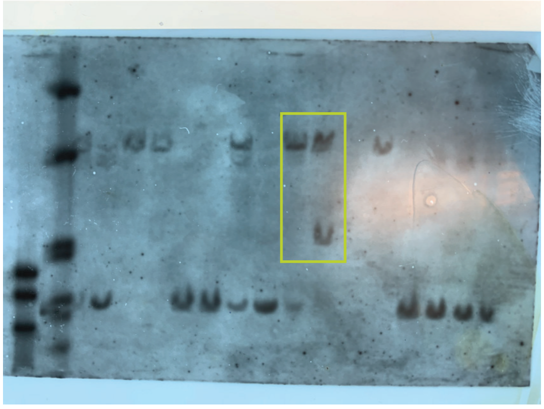

**Figure 5, Source Data 1.** Original membranes corresponding to Figure 5, panel C. The relevant lanes are outlined in yellow.
