# Supplementary material for: Shc1 cooperates with Frs2 and Shp2 to recruit Grb2 in FGF-induced lens development
Source: eLife. 2025 May 6;13:RP103615. doi: 10.7554/eLife.103615 (PMC12055001; doi:10.7554/eLife.103615)
Supplement: Figure 5—source data 4. [file elife-103615-fig5-data4.zip › Figure 5H Source data 4.pdf]

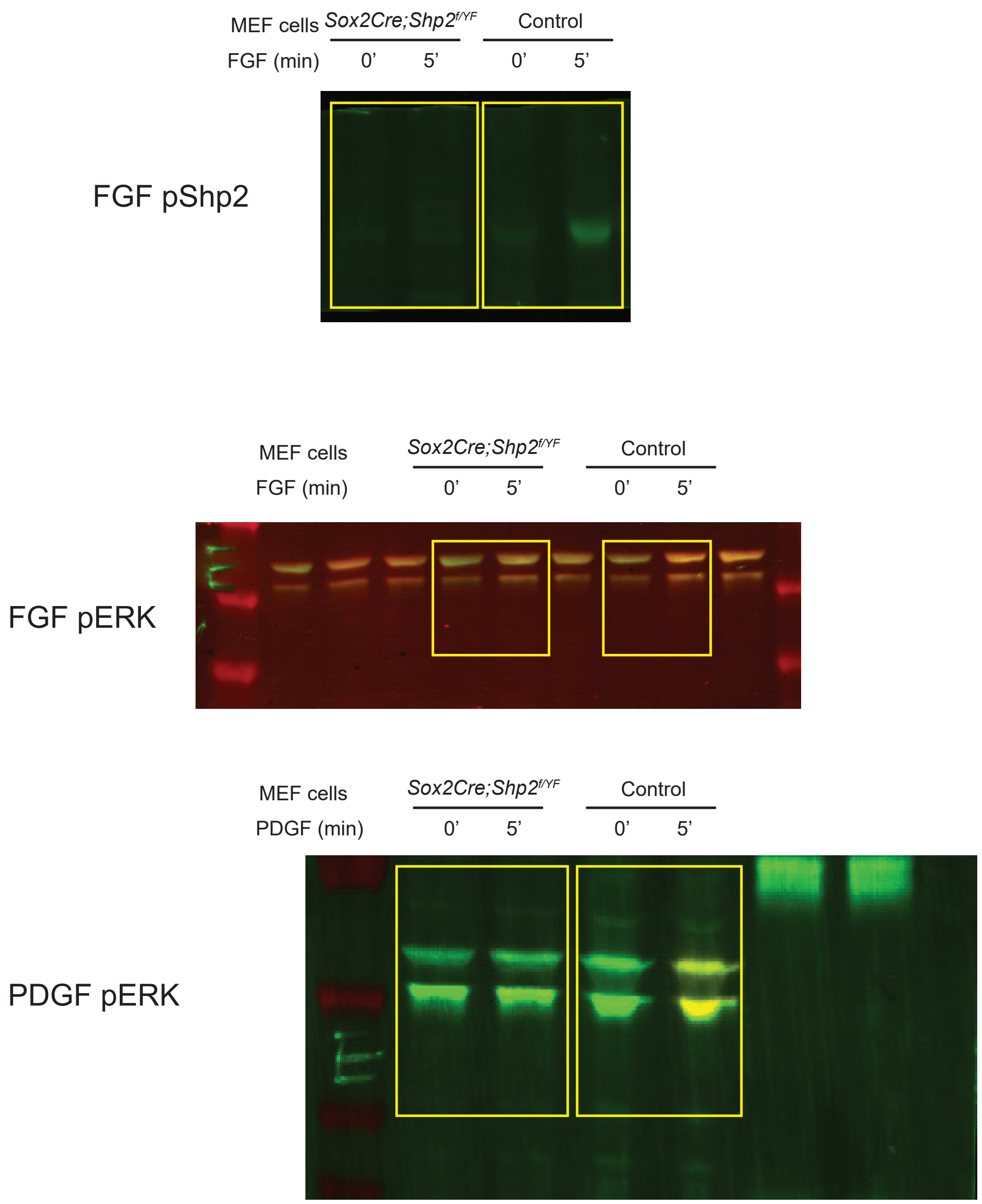

Figure 5, Source Data 4. Original membranes corresponding to Figure 5, panel H. The relevant lanes are outlined in yellow.
