# Supplementary material for: Shc1 cooperates with Frs2 and Shp2 to recruit Grb2 in FGF-induced lens development
Source: eLife. 2025 May 6;13:RP103615. doi: 10.7554/eLife.103615 (PMC12055001; doi:10.7554/eLife.103615)
Supplement: Figure 7—source data 2. [file elife-103615-fig7-data2.zip › Figure 7A Source data 2.pdf]

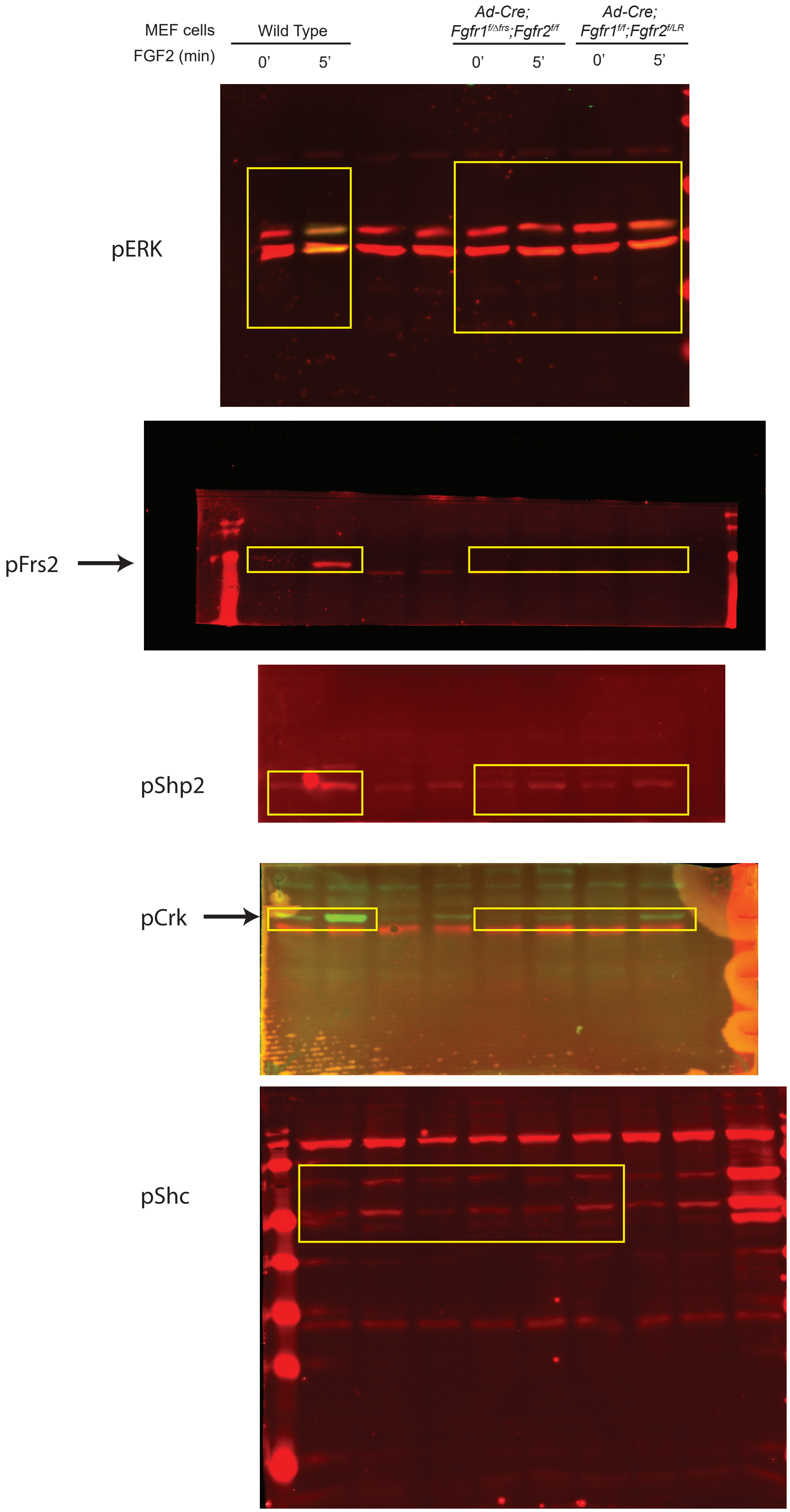

Figure 7, Source Data 2. Original membranes corresponding to Figure 7, panel A. The relevant lanes are outlined in yellow.
